# Effects of Lysophosphatidylcholine on Jejuna Morphology and Its Potential Mechanism

**DOI:** 10.3389/fvets.2022.911496

**Published:** 2022-06-20

**Authors:** Xiaofeng Li, Abdel-Moneim Eid Abdel-Moneim, Noura M. Mesalam, Bing Yang

**Affiliations:** ^1^College of Animal Science, Anhui Science and Technology University, Huainan, China; ^2^Department of Biological Applications, Nuclear Research Center, Egyptian Atomic Energy Authority, Abu-Zaabal, Egypt

**Keywords:** gene, signaling pathway, lysophosphatidylcholine, jejuna, broiler

## Abstract

Lysophosphatidylcholine (LPC) plays a vital role in promoting jejuna morphology in broilers. However, the potential mechanism behind LPC improving the chicken jejuna morphology is unclear. Therefore, the present study was designed to reveal the important genes associated with LPC regulation in birds' jejuna. Thus, GSE94622, the gene expression microarray, was obtained from Gene Expression Omnibus (GEO). GSE94622 consists of 15 broiler jejuna samples from two LPC-treated (LPC500 and LPC1000) and the control groups. Totally 98 to 217 DEGs were identified by comparing LPC500 vs. control, LPC1000 vs. control, and LPC1000 vs. LPC500. Gene ontology (GO) analysis suggested that those DEGs were mainly involved in the one-carbon metabolic process, carbon dioxide transport, endodermal cell differentiation, the positive regulation of dipeptide transmembrane transport, cellular pH reduction, and synaptic transmission. Kyoto Encyclopedia of Genes and Genomes (KEGG) analysis indicated the DEGs were enriched in NOD-like receptor (NLR), RIG-I-like receptor (RILR), Toll-like receptor (TLR), and necroptosis signaling pathway. Moreover, many genes, such as *RSAD2, OASL, EPSTI1, CMPK2, IFIH1, IFIT5, USP18, MX1, and STAT1* might be involved in promoting the jejuna morphology of broilers. In conclusion, this study enhances our understanding of LPC regulation in jejuna morphology.

## Introduction

Lysophosphatidylcholine (LPC), a kind of bioactive lipid, has a robust antimicrobial and immunomodulatory potentials in broilers (1–5). The potent antimicrobial activity of LPC has been documented (1–3). In the *in vitro* study of Yadav et al. (1), the combination of LPC and polymyxin B inhibited the growth of Salmonella and some Gram-negative. In addition, LPC was substantially more effective to induce membrane permeability in Gram-positive bacteria than in Gram-negative counterparts. The bactericidal effect of LPC also enhanced gentamicin sensitivity in resistant Methicillin-resistant *Staphylococcus aureus* strains and markedly alleviated the burden from intracellular bacteria in the liver and spleen (2, 3). Furthermore, LPC improved phagosome maturation to control *Salmonella Typhimurium* (*S. Typhimurium*) growth by activating the NFκB pathway induced by active oxygen species (ROS) (3). In LPC-treated cells, the expression of phagosome maturation markers (including *LAMP1* and *EEA1*) and cleaved cathepsin D, and ROS production were remarkably improved during *S. Typhimurium* infection (3). LPC also enhanced the production of gamma interferon by engaging naive T cells and induced the release of chemokines and IL-8 (4).

Parra-Millan et al. reported that LPC could trigger innate and specific humoral-mediated immunity as LPC administration regulated the immune response in the prognosis of ceftazidime-resistant *Pseudomonas*-induced infections (6). Moreover, in monocyte-derived dendritic cells, LPC could upregulate the *CD86, HLA-DR*, and *CD40* genes expression (6). LPC increased the relative weights of bursa and thymus, and improved antibody production titers against Newcastle disease virus and sheep red blood cells in broilers (7).

Importantly, previous investigations also revealed that LPC had the ability to improve intestinal morphology, nutrients digestion and absorption, and growth performance in broilers (6–10). Nutautaite et al. documented significant improvement in intestinal villus height, average daily gain (ADG), and the contents of isovaleric and butyric acid in broilers treated with LPC (8). LPC addition reduced the crypt depth, increased the jejunal villi height, and improved the ratio of villi height to crypt depth in the jejuna and duodena of chicken. LPL promoted the growth performance and nutrient utilization (9). Zhang et al. reported that dietary LPC increased body weight gain and the digestibility of C16:0, C18:1n7, C18:2, C18:3n3, and C18:1n9 (10). Furthermore, LPC decreased the average daily feed intake, increased ADG and feed conversion ratio (7). LPC improves broilers' performance by upregulating the expression of amino acids and cholesterol transporter genes in enterocytes and increasing the fat digestibility and the intake of cholesterol and amino acids (6).

Nevertheless, the mechanism by which LPC improves the jejuna morphology in broilers is unclear. Therefore, we obtained the microarray data of broiler chickens' jejuna treated with or without LPC from the Gene Expression Omnibus dataset (GEO; https://www.ncbi.nlm.nih.gov/geo/) and identified differentially expressed genes (DEGs) in birds' jejuna, aiming to explore the potential mechanism behind the regulation of LPC on the jejuna morphology in broilers.

## Materials and Methods

### Ethics Statement

This study protocol was approved by Anhui Science and Technology University (Bengbu, China) Institutional Animal Care and Use Committee (ECASTU-2015-P08).

### Animals, Feed, and Tissue Collection

Seventy-five newly hatched Cobb 500 male broilers were divided into three groups, including the control, LPC500, and LPC1000 groups, with five replications of five chicks each (11). The control was provided with the basal diet, and the LPC500 and LPC1000 groups were provided with the basal diet adding 500 g/T and 1,000 g/T LPC, respectively (11). The experimental time lasted for 4 weeks. Ingredients and nutrient levels of the basal diet were shown in Supplementary Table 1. All birds were individually weighed weekly and placed in a room with adjoining floor pens (11). On the 10th day of the experiment, five chicks were randomly chosen from each group and killed via cervical dislocation. Pieces with approximately 10 cm in length were collected from the middle of jejuna (11).

### RNA Extraction and Microarray Analysis

Based on the effects of LPC on chicken jejuna morphology (Supplementary Table 2), Approximately 50 mg of jejunal mucosa was homogenized using Tri Reagent (11). Total RNA was extracted using Directzol RNA columns, and RNA integrity, quality, and purity were assessed (11). Samples with RNA integrity number (RIN) > 8.7 were used for the subsequent analysis. Microarray analysis was performed with the chicken genome 1.0 array (11). The data of jejuna gene expression were deposited in GEO (accession number: GSE94622) (11).

### Microarray Data

The gene expression microarray, GSE94622, was downloaded from the GEO dataset. GSE94622 consisted of LPC1000-treated (n = 5; GSM2479496, GSM2479497, GSM2479513, GSM2479530, and GSM2479531), LPC500-treated (n = 5; GSM2479493, GSM2479510, GSM2479511, GSM2479526, and GSM2479527) and the control (n = 5; GSM2479490, GSM2479491, GSM2479506, GSM2479507, and GSM2479523) chicken jejuna samples obtained at the 10^th^day of experiment.

### Data Processing

To identify the DEGs in the jejuna samples between the LPC-treated groups and the control, GEO2R (http://www.ncbi.nlm.nih.gov/geo/geo2r) software was used the data from GSE94622. Genes with|log_2_Fold Change (FC)| > 1and *P* < 0.05 were considered as the DEGs. The probe sets without Entrez gene annotation were deleted.

### Analysis of KEGG and Genetic Ontology for DEGs

KOBAS 3.0(http://kobas.cbi.pku.edu.cn/kobas3/genelist/) was used to analyze the signaling pathway for DEGs. As to the Genetic ontology (GO) analysis, DEGs were analyzed with DAVID (https://david.ncifcrf.gov/).

### Protein Classification and Reactome Analysis for DEGs

Protein class and Reactome analysis for DEGs were performed with PANTHER classification system (http://pantherdb.org/) and KOBAS 3.0, respectively.

### Protein-Protein Interaction Network

Protein-Protein Interaction (PPI) network was analyzed with the STRING database (https://string-db.org/) and further visualized with Cytoscape 3.8.0 (http://www.cytoscape.org/).

### Hub Genes and Their Functions

CytoHubba software (http://apps.cytoscape.org/apps/cytohubba) was employed to reveal hub genes from the PPI network, then the functions of hub genes were summarized using GeneCards (https://www.genecards.org/), NCBI database (https://www.ncbi.nlm.nih.-gov/), and previous literature.

## Results

### Outline of Transcripts and Genes in Broilers Jejuna

**Figure 1:**
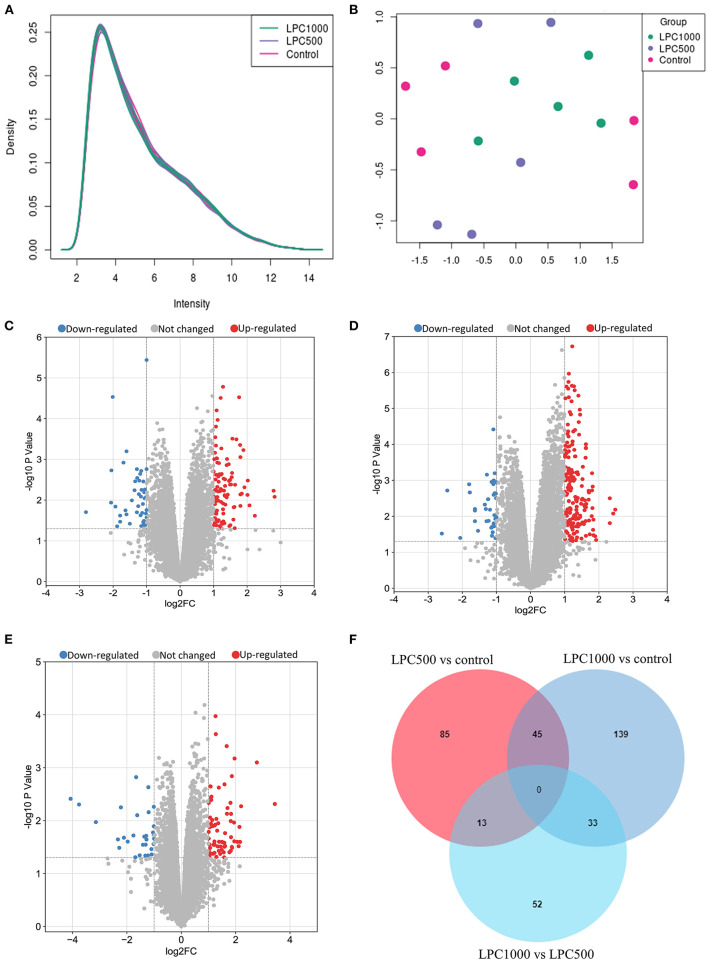
Profile of Transcripts and Genes in Broilers Jejuna Treated with and without LPC. **(A)** Transcripts expression density; **(B)** UMAP; **(C–E)** volcano plot of DEGs identified by three comparisons (LPC500 and LPC1000 vs. control and LPC1000 vs. LPC500, respectively). The red gray, and blue spots represent the upregulated, unchanged and downregulated genes, respectively. **(F)** Venn diagrams for the DEGs identified in the three ways of comparisons mentioned above.

Totally 38,535 transcripts and 14,086 genes were identified in the jejuna treated with or without LPC. UMAP and transcripts expression density are shown in Figures 1A,B. Figures 1C–E represents the volcano plots for DEGs in the comparisons of LPC500 and LPC1000 vs. control and LPC1000 vs. LPC500, respectively. Figure 1F represents the Venn diagram of the three comparisons.

As shown in Supplementary Table 3, a total of 147 to 306 differentially expressed transcripts (DETs), 98 to 217 DEGs were identified by three ways of comparisons (LPC500 vs. control, LPC1000 vs. control, and LPC1000 vs. LPC500). Compared with the control, 236 transcripts and 179 genes were upregulated, whereas 70 transcripts and 38 genes were downregulated in LPC1000-treated jejuna (Supplementary Files 1, 2); 145 transcripts and 99 genes were upregulated, whereas 70 transcripts and 44 genes were downregulated in LPC500-treated jejuna (Supplementary Files 3, 4). Forty of the 45 common DEGs of the two comparisons (LPC1000 vs. control and LPC500vs.control) were shown in Supplementary Table 4. The top 20 up-and downregulated genes in three comparisons (LPC500 and LPC1000 vs. control and LPC1000 vs. LPC500) were revealed in Supplementary Tables 5–10, respectively.

### GO Analysis for DEGs

**Figure 2:**
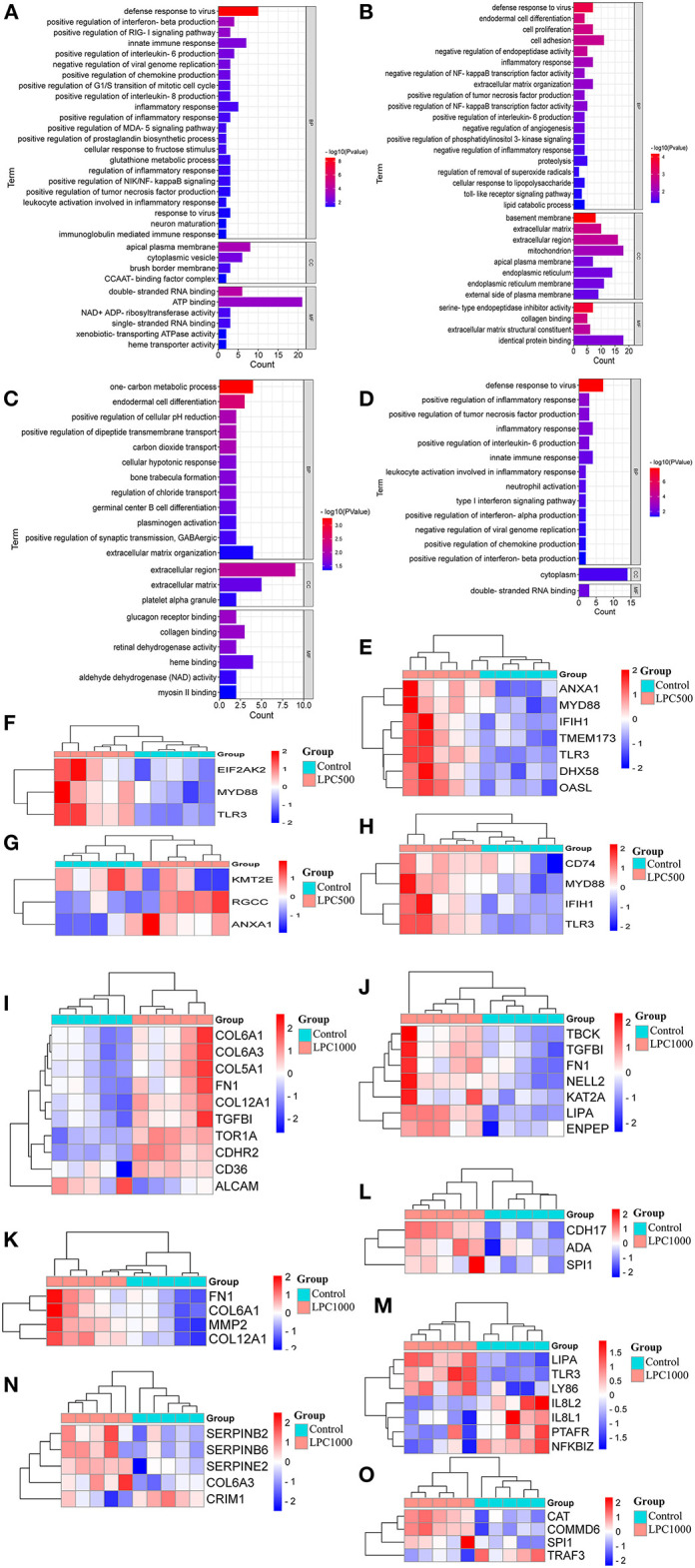
GO Enrichment for DEGs in Broilers Jejuna Treated with and without LPC. **(A–C)** GO enrichment for DEGs identified in the three comparisons (LPC500 and LPC1000 vs. control and LPC1000 vs. LPC500, respectively); **(D)** GO enrichment for the common DEGs in the two comparisons (LPC500 and LPC1000 vs. control);The heatmaps for DEGs in innate immune response **(E)**, the positive regulation of chemokine production **(F)**, the mitotic cell cycle G1/S transition **(G)**, IL-6 production **(H)**,cell adhesion **(I)**, cell proliferation **(J)**, endodermal cell differentiation **(K)**, germinal center B cell differentiation **(L)**, inflammatory response **(M)**, the negative regulation of endopeptidase activity **(N)**, and the negative regulation of NFκB transcription factor activity **(O)**, respectively.

To reveal the biological processes involved in the regulation of LPC on broiler jejuna heath, GO functional enrichment of DEG sin the three comparisons, including LPC1000 andLPC500 vs. control, and LPC1000 vs. LPC500were illustrated in Figures 2A–C and Supplementary Files 5–7, respectively.

Forty-five common DEGs of two comparisons (LPC1000 and LPC500 vs. control) in the chicken jejuna participated in defense response to viruses, the positive regulation of tumor necrosis factor production, inflammatory response, interleukin-6 production, and innate immune response; neutrophil activation; type I interferon; the negative regulation of viral genome replication, chemokine, interferon-alpha, interferon-beta production (Figure 2D and Supplementary File 8). In addition, Figures 2E–O represents the heatmaps for DEGs in innate immune response; the positive regulation of chemokine production; the positive regulation of G1/S transition of the mitotic cell cycle, the positive regulation of interleukin-6 production, cell adhesion, cell proliferation, endodermal cell differentiation, germinal center B cell differentiation, inflammatory response; the negative regulation of endopeptidase activity; and the negative regulation of NFκB transcription factor activity, respectively.

### KEGG Enrichment for DEGs

**Figure 3:**
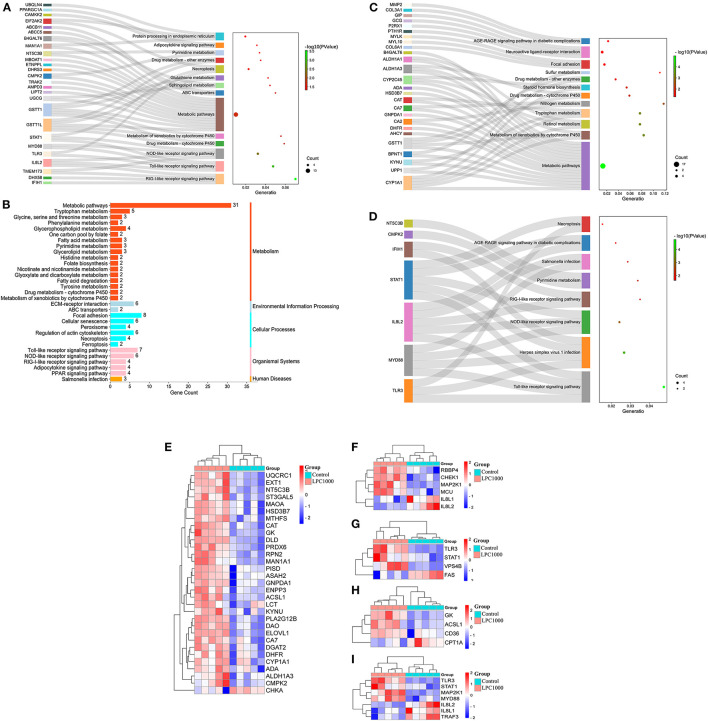
KEGG Enrichment for DEGs in Broilers Jejuna Treated with and without LPC. **(A–C)** KEGG enrichment for DEGs identified by three comparisons (LPC500 and LPC1000 vs. control and LPC1000 vs. LPC500, respectively); **(D)** KEGG enrichment for the common DEGs identified by two comparisons (LPC500 and LPC1000 vs. control); Heatmaps for DEGs in metabolic pathways **(E)**, cellular senescence **(F)**, necroptosis **(G)**, PPAR **(H)**, and TLR signaling pathway **(I)**, respectively.

To discover the signaling pathways related to the regulation of LPC on broiler jejuna heath, KEGG enrichment for DEGs in the three comparisons, including LPC500 vs. control, LPC1000 vs. control, and LPC1000 vs. LPC500, is shown in Figures 3A–C and Supplementary Files 9, 10, respectively. Moreover, the common DEGs of two comparisons (LPC500 vs. control and LPC1000 vs. control) mainly participated in TLR, NLR, RILR, pyrimidine metabolism, AGE-RAGE, and necroptosis signaling pathways (Figure 3D and Supplementary File 12). In addition, Figures 3E–I reveals the expression outline for DEGs in metabolic pathways, cellular senescence, necroptosis, PPAR, and TLR signaling pathway, respectively.

### Reactome Enrichment for DEGs

**Figure 4:**
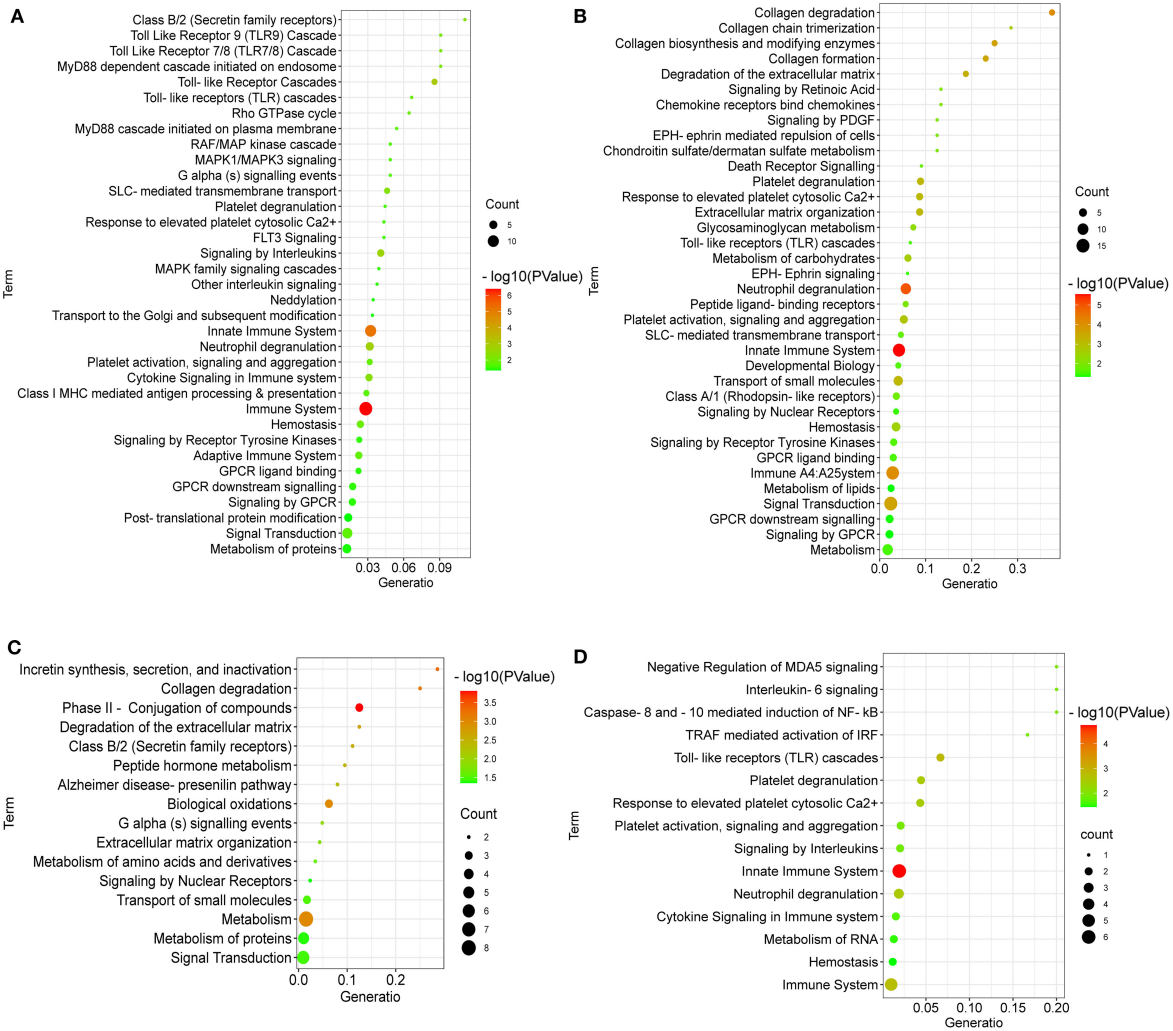
Reactome Enrichment for DEGs in Broilers Jejuna treated with and without LPC. **(A–C)** Reactome enrichment for DEGs identified by three comparisons (LPC500 and LPC1000 vs. control and LPC1000 vs. LPC500, respectively); **(D)** Reactome enrichment for the common DEGs in two comparisons (LPC500 and LPC1000 vs. control).

To further reveal the pathways related to the regulation of LPC on broiler jejuna heath, Reactome enrichment for DEGs in the three comparisons, including LPC500 vs. control, LPC1000 vs. control, and LPC1000 vs. LPC500, are shown in Figures 4A–C, respectively. In addition, the common DEGs of two comparisons (LPC500 and LPC1000 vs. control) mainly taken part in the innate immune system; cytokine signaling in the immune system; metabolism of RNA, hemostasis; caspase-8 and−10 mediated induction of NF-Kb; interleukin-6 signaling; negative regulation of MDA5 signaling; TRAF mediated activation of IRF; and interleukin-20 family signaling (Figure 4D).

### Protein Classification of DEGs

**Figure 5:**
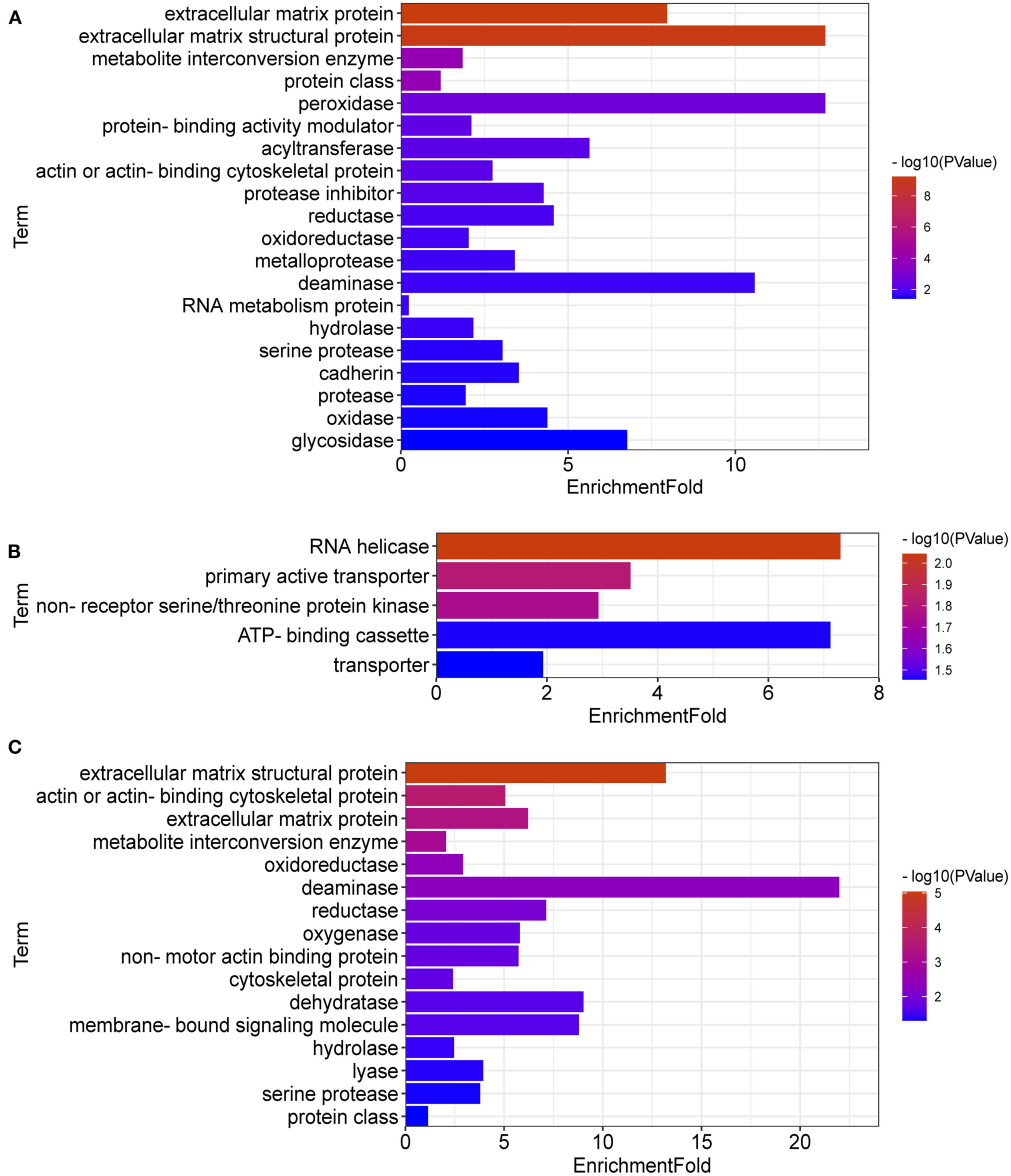
Protein Classification for DEGs in Broilers Jejuna Treated with and without LPC. **(A–C)** Protein classification for DEGs identified by three comparisons (LPC1000 and LPC500 vs. control, and LPC1000 vs. LPC500, respectively).

DEGs between LPC1000 and the control groups might play an important role in extracellular matrix protein, extracellular matrix structural protein, metabolite interconversion enzyme, protein class, peroxidase, protein-binding activity modulator, acyltransferase, actin or actin-binding cytoskeletal protein, protease inhibitor, reductase, oxidoreductase, metalloprotease, deaminase, RNA metabolism protein, hydrolase, serine protease, cadherin, protease, oxidase and glycosidase (Figure 5A). DEGs between LPC500 and the control groups might play an important role in RNA helicase, primary active transporter, non-receptor serine/threonine-protein kinase, ATP-binding cassette, transporter (Figure 5B). DEGs between LPC1000 and LPC500 groups might play an important role in extracellular matrix structural protein, metabolite interconversion enzyme, oxidoreductase, deaminase, reductase, oxygenase, dehydratase, membrane-bound signaling molecule, hydrolase, lyase, and serine protease (Figure 5C).

### PPI Network

**Figure 6:**
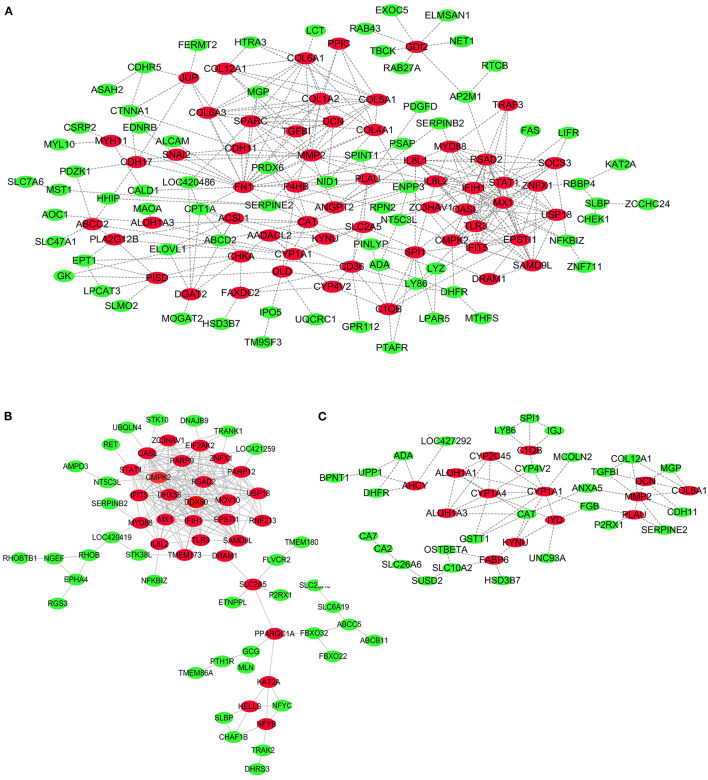
PPI Network for DEGs in Broilers Jejuna Treated with and without LPC. **(A–C)** PPI network for DEGs identified by three comparisons (LPC1000 and LPC500 vs. control, and LPC1000 vs. LPC500, respectively).

To further explore key genes, PPI networks of DEGs in the three comparisons, including LPC500 andLPC1000 vs. control, and LPC1000 vs. LPC500, are shown in Figures 6A–C, respectively.

### Hub Genes and Their Function

**Figure 7:**
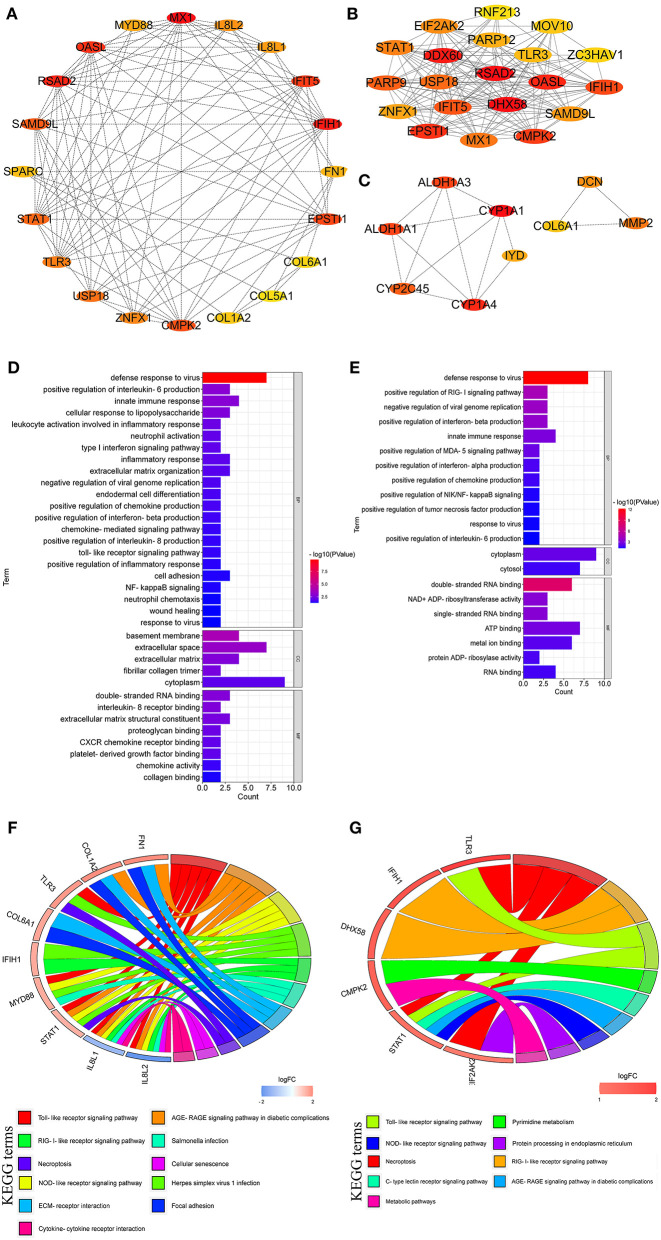
Hub genes linked to the regulation of LPC on the jejuna morphology in broiler. **(A–C)** Hub genes identified by three comparisons (LPC1000 and LPC500 vs. control, and LPC1000 vs. LPC500, respectively); **(D,E)** GO enrichment for hub genes identified by two comparisons (LPC1000 and LPC500 vs. control, respectively); and **(F,G)** KEGG enrichment for hub genes identified by two comparisons (LPC1000 and LPC500 vs. control, respectively).

As shown in Figure 7A, the top 20 hub genes from DEGs between LPC1000 and control groups included *MX1, IFIH1, RSAD2, OASL, IFIT5, EPSTI1, CMPK2, SAMD9L, STAT1, USP18, TLR3, ZNFX1, IL8L2, IL8L1, MYD88, FN1, SPARC, COL1A2, COL6A1*, and *COL5A1*. The top 20 hub genes from DEGs between LPC500 and control groups included *DHX58, RSAD2, DDX60, OASL, EPSTI1, CMPK2, IFIH1, IFIT5, PARP9, USP18, MX1, STAT1, EIF2AK2, SAMD9L, ZNFX1, PARP12, TLR3, MOV10, ZC3HAV1*, and *RNF213* (Figure 7B). The top nine hub genes from DEGs between LPC1000 and LPC500 groups included *CYP1A1, CYP1A4, ALDH1A1, ALDH1A3, CYP2C45, MMP2, DCN, IYD*, and *COL6A1* (Figure 7C).

GO enrichment for the top 20 hub genes from DEGs between LPC1000, and the control groups suggested that these genes participated in leukocyte activation involved in TLR; cell adhesion; neutrophil activation; wound healing; type I interferon; inflammatory response; extracellular matrix organization; NFκB inflammatory response; endodermal cell differentiation, chemokine-mediated; neutrophil chemotaxis, the response to virus; and the positive regulation of inflammatory response, and the production of IL-6, IL-8, chemokine and interferon-beta (Figure 7D). GO enrichment for the top 20 hub genes from DEGs between LPC500 and control groups suggested that these genes participated in the positive regulation of RIG-I, MDA-5, NIK/NFκB signaling and the productions of chemokine, interferon-alpha, tumor necrosis factor, et al. (Figure 7E).

KEGG enrichment suggested the top 20 hub genes from DEGs between LPC1000 and control were involved in multiple signaling pathways, including TLR; necroptosis; cellular senescence; AGE-RAGE; focal adhesion; NLR; and RILR (Figure 7F). KEGG enrichment indicated that the top 20 hub genes from DEGs between LPC500 and control groups participated in many signaling pathways, including TLR; necroptosis; pyrimidine metabolism; AGE-RAGE; RILR; C-type lectin receptor; NLR; protein processing in the endoplasmic reticulum; and metabolic pathways (Figure 7G).

## Discussion

### Hub Genes by Which LPC Increased Jejuna Morphology in Broilers

In the present study, many hub genes, such as *RSAD2, OASL, EPSTI1, CMPK2, IFIH1, IFIT5, USP18, MX1*, and *STAT1*, might be concentrated in the regulation of LPC on the chicken jejuna morphology. *RSAD2* (Radical s-adenosyl methionine domain containing 2) encoded a vital enzyme for innate immune responses as it was expressed in multiple kinds of cells in response to inflammatory stimuli (including viral infection). For instance, Wiedemann et al. reported that *RSAD2* participated in the NK cells' adaptive behavior after viral infection (12). In infected 293T cells, *RSAD2* could restrict the measles virus (MV) infection at the virus release stage, However, in SR-B2 cells, the transduction with *RSAD2* expression *in vitro* or *in vivo* impaired the MV release (13). *RSAD2* also catalyzed the transformation of cytidine triphosphate (CTP) to its analog ddhCTP, which inhibited the activity of NAD^+^-dependent enzymes, including *GAPDH* (14). Therefore, *RSAD2* might control the cell response to inflammatory stimuli, including viral infection, by regulating cell metabolism (15).

*OASL* (2′-5′-Oligoadenylate Synthetase Like) exerts various effects on RNA and DNA viruses by improving RIG-I-induced IFN induction and suppressing cGAS-induced IFN production, respectively (16). In CD4^+^ T cells, *OASL* gene upregulation could increase the expressions of TET1, CD40L, and CD70, the hydroxymethylation levels, and the aberrant cell activation via IRF1 signaling. Moreover, *IRF1* could regulate *TET1* expression by binding to its promoter (17).

*EPSTI1*, named epithelial-stromal interaction 1, was widely expressed in immune cell types and had a vital role in immune privilege and function. In our study, *EPSTI1* was significantly up-regulated in chicken jejuna from LPC500 and LPC1000 groups compared to the control which was consistent with Kim et al. who reported that *EPSTI1* was highly expressed in macrophages activated by the exposure to lipopolysaccharide (LPS) and IFNγ (18). Macrophage polarization was important for the resistance to various infections (18). Additionally, *EPSTI1* was of great importance for antiviral activity mediated by IL-28A. *EPSTI1* actually restrained HCV replication without interferon, and *EPSTI1* knockdown resulted in viral enhancement. EPSTI1 could activate the *PKR* gene promoter and induce multiple *PKR*-dependent genes (such as *OAS1, IFIT1*, and *IFN*β) responsible for the antiviral activity of *EPSTI1* (14).

*CMPK2*, known as cytidine/uridine monophosphate kinase 2, was a vital factor for innate immunity and infection. *CMPK2* played a critical role in dengue virus (DENV)-induced mitochondrial oxidative stress, cytokine release, and mitochondrial DNA (mtDNA) release to the cytosol. CMPK2 depletion could suppress the DENV-induced cell migration, TLR9 activation, and inflammasome pathway, and the increasing viral production (19). CMPK2 mediated the antiviral activity (19). In the present study, *CMPK2* was obviously up-regulated in chicken jejuna from LPC-treated groups which agree with the report that *CMPK2* was involved in mtDNA synthesis and antiviral immunity in animals (20). Multiple tissues exist for *CMPK2* expression, and bacterial infection could upregulate *CMPK2* expression in a time-dependent manner. *CMPK2* induced NLRP3 activation and mtDNA synthesis. *CMPK2* overexpression protected the intestinal barrier and hindered bacterial colonization (20).

*IFIH1* (Interferon induced with helicase C domain 1) is an important virus cytosolic sensor encoding MDA5 protein. *IFIH1* variants were significantly enriched in children with Very Early Onset Inflammatory Bowel Disease (VEOIBD) compared to the control (21). Partial or complete MDA5 protein deficiency was linked to VEOIBD with variable expressivity and penetrance, implying a vital role for impaired intestinal viral sensing in the pathogenesis of Inflammatory Bowel Disease (IBD) (21). *IFIH1* also induced an antiviral Type I interferon (IFN) state. *IFIH1* overexpression increased Type I IFN activity. *FIH1* mutation might be related to the inflammatory cell infiltration into the intestinal epithelium and the thickened states and edema of the small intestine and colon (22). *IFIH1* was also the regulatory factor for facilitating M1 macrophage polarization by activating the*IRF3* gene. Viral RNAs stimulated the activation of IFIH1-IRF3. IFIH1-IRF3 activation induced by LPS was in a MyD88-dependent manner (22).

*IFITs* played a vital role in maintaining homeostasis and regulating immune responses. In HEK293T cells, *IFIT5* functioned as the negative regulatory factor in the IFN pathway. *IFIT5* might inhibit *IFN*β promoter activities by targeting *IRF3*. IFIT5 reduced the IRF3 protein phosphorylation and restrained the *IRF3* nuclear translocation (23).

*USP18*, a mitochondria-localized deubiquitinase, specifically interacted with mitochondrial antiviral signaling protein (MAVS) and promoted K63-linked polyubiquitination and subsequent MAVS aggregation. *USP18* upregulated the IFN production and expression following virus infection (24). Mice with *USP18* deficiency were more susceptible to RNA virus infection. USP18, which served as a scaffold protein, could enhance the TRIM31 re-localization and facilitate the interactions between MAVS and TRIM31 in mitochondria (25). *USP18* was the negative regulatory factor for IFN signaling, and DENV infection significantly increased *USP18* expression (26).

*STAT1*, aka signal transducer and activator of transcription 1, might play a central role in the intestinal epithelium heath in a caspase-8-dependentmanner because it was located at the crossroad of multiple cell death-associated signaling pathways. In *CASP8*-deficient mice, *STAT1* activation in epithelium aggravated the sensibility toward bacterial-induced enteritis, intestinal inflammation, and lethality. *STAT1* depletion abrogated the intestinal barrier breakdown, epithelial cell loss, cell death, and systemic infection (27).

As discussed above, the hub genes, including *RSAD2, OASL, EPSTI1, CMPK2, IFIH1, IFIT5, USP18, MX1*, and *STAT1*, might be closely related to intestinal inflammation and infection, which affected the intestinal morphology characterized by the obvious changes in villus height and width, and crypt depth (28–31).

### Signaling Pathways by Which LPC Increased the Jejuna Morphology in Broilers

In this research, we found that LPC regulated jejuna morphology via multiple signaling pathways, such as toll-like receptor (TLR), nod-like receptor (NLR), and necroptosis pathways. Previous studies indicated that TLR, NLR, and RILR were a variety of pathogen pattern recognition receptors involved in maintaining intestinal morphology, homeostasis and health (32–41). *TLR* mediated the inflammatory responses of intestinal mucosa macrophages to resist the pathogen invasion (32). *TLR* stimulation was enough to increase the MTDH expression, but MTDH depletion was also enough to restrain macrophages from producing inflammatory cytokines induced by TLR. Moreover, TLR could induce NFκB and MAPK signaling which were closely linked to the alleviated inflammatory reaction and the improved jejunal morphology after LPS stimulation (32, 33).

*TLR2* might regulate several essential enteric physiological functions and pathological processes, including innate immune, peristaltic reflexes, intestinal serotonergic response, enteropathogenic infections, and gastrointestinal fluid absorption or secretion (34). *TLR2* activation might involve in the inflammatory response of the neuroendocrine cells. *TLR2* inhibition alleviates the oxidative stress and tissue damage of the intestinal tract, characterized by reduced pro-inflammatory cytokines production and restored SERT activity (34). When exposed to commensal or pathogenic bacteria, the intestinal innate immune cells would release the TLRs ligands (35). TLR signaling pathway mediated the intestinal microbiota disorder and contributed to the intestinal Graft-vs.-host disease development (35).

NLRs, a kind of cytosolic pattern-recognition receptors, played an important role in mucosal immune defense, intestinal infections, maintaining gut homeostasis and morphology, and shaping the microbiota. The current study showed that DEGs in chicken jejuna between LPC-treated groups and the control enriched in NLR signaling pathway which agree with the research that NLRs might function as the intestinal barrier guardian, given their association with NOD2 and inflammatory bowel disease (36). NLRs mediated inflammatory cell pyroptosis, caspase-1 activation. NLRs might be involved in the common gastrointestinal bacterial pathogen infection in the small intestine (37). NLRs also regulate intestinal microbiota. NLR proteins, kind of cytoplasmic microbial sensors, were involved in various intestinal disorders, such as inflammatory bowel diseases (38). NLRs mediated gut protection by regulating the intricate interaction among immune, stromal, and epithelial cells. NLRs-mediated protection often needed the assistance of STAT3, MAPK, and NFκB pathways (39). Inflammation-related signaling pathways, including RILR, TLR, NLRs, NFκB, Jak-STAT, and TNF, mediated the intestinal epithelial cell (IEC) infection and severe inflammation induced by transmissible infection gastroenteritis virus (40).

Necroptosis, a kind of programmed cell death, was recently discovered. It combined the characteristics of necrosis and apoptosis, which was important in the intestinal injury and morphology. In our study, DEGs in chicken jejuna between LPC-treated groups and the control enriched in necroptosis signaling pathway which was consistent with the research that LPS resulted in typical cell necrosis characterized by enhanced expression of necroptosis protein (including RIP1, RIP3, MLKL, PGAM5, DRP1, and HMGB1) and the impairment of jejunal morphology in pig (41). Also, necroptosis dysregulation prevented the resolution of intestinal inflammation. E-type prostanoid receptor 4 suppressed the necroptosis by converging on receptor-interacting protein kinase 1 to reduce TNF-induced activation and the necroptosis effector membrane translocation in human and mouse IECs (42).

## Conclusions

In conclusion, this study provides a valuable resource for identifying genes and signaling pathways associated with the regulation of LPC on the jejuna morphology in broiler.

## Data Availability Statement

Publicly available datasets were analyzed in this study. This data can be found here: https://www.ncbi.nlm.nih.gov/geo/query/acc.cgi?acc=GSE94622.

## Ethics Statement

The animal study was reviewed and approved by Anhui Science and Technology University (Bengbu, China) Institutional Animal Care and Use Committee.

## Author Contributions

BY analyzed the results and prepared the tables and figures. XL wrote the manuscript. A-MA-M and NM revised the manuscript. All authors contributed to the article and approved the submitted version.

## Funding

This study was funded by the Talent Introduction Program of Anhui Science and Technology University (No. DKYJ202003).

## Conflict of Interest

The authors declare that the research was conducted in the absence of any commercial or financial relationships that could be construed as a potential conflict of interest.

## Publisher's Note

All claims expressed in this article are solely those of the authors and do not necessarily represent those of their affiliated organizations, or those of the publisher, the editors and the reviewers. Any product that may be evaluated in this article, or claim that may be made by its manufacturer, is not guaranteed or endorsed by the publisher.
