# Supplementary material for: Effects of Lysophosphatidylcholine on Jejuna Morphology and Its Potential Mechanism
Source: Front Vet Sci. 2022 Jun 20;9:911496. doi: 10.3389/fvets.2022.911496 (PMC9252431; doi:10.3389/fvets.2022.911496)
Supplement: Supplementary file 1 [file Table_1.docx]

Supplemental Table 1. Ingredients and nutrient content of basal diet (Brautigan et al, 2017)

| Items | Diets | |
| --- | --- | --- |
|  | Starter/% | Grower/% |
| Ingredients |  |  |
| Corn | 55.07 | 59.26 |
| Soybean meal 48 | 30.10 | 22.87 |
| Poultry byproduct meal | 3.00 | 4.00 |
| DDGS | 3.18 | 6.00 |
| Stabilized fat | 2.50 | 4.80 |
| Limestone | 1.00 | 0.84 |
| Dicalcium phosphate | 1.04 | 0.70 |
| Sodium chloride | 0.37 | 0.32 |
| DL-Methionine | 0.28 | 0.23 |
| Lysine-HCl | 0.20 | 0.21 |
| Threonine | 0.07 | 0.04 |
| Phytase/ftu/kg | 500 | 500 |
| Choline chloride 60 | 0.10 | 0.10 |
| Mineral premix | 0.31 | 0.31 |
| Vitamin premix | 0.31 | 0.31 |
| Soy oil | 1.50 |  |
| Nutrient content |  | |
| Metabolic energy/Mcal/kg | 3.10 | 3.18 |
| Crude protein | 21.84 | 20.06 |
| Available phosphorus | 0.35 | 0.32 |
| Calcium | 0.80 | 0.74 |
| Methionine + cysteine | 0.88 | 0.80 |
| Threonine | 0.77 | 0.69 |
| Lysine | 1.18 | 1.05 |

Supplemental Table 2. Effects of LPC on chicken jejuna morphology (Brautigan et al, 2017)

| Items | Groups | | | SEM | *P* Value |
| --- | --- | --- | --- | --- | --- |
|  | Control | LFT500 | LFT1000 |  |  |
| Villus width/μm | 140.10^b^ | 142.09^b^ | 179^a^ | 6.24 | <0.001 |

Supplemental Table 3. The transcripts and genes significantly up- and down-regulated in LPC-treated jejuna

| Items | Comparisons | Total | Up-regulated | Down-regulated |
| --- | --- | --- | --- | --- |
| Transcripts | LPC1000 vs control | 306 | 236 | 70 |
|  | LPC500 vs control | 215 | 145 | 70 |
|  | LPC1000 vs LPC500 | 147 | 101 | 46 |
| Genes | LPC1000 vs control | 217 | 179 | 38 |
|  | LPC500 vs control | 143 | 99 | 44 |
|  | LPC1000 vs LPC500 | 98 | 69 | 29 |

Supplemental Table 4. Common DEGs in the two comparisons (LPC500 and LPC1000 vs. control)

| Gene symbol | Full name | Functions |
| --- | --- | --- |
| *RSAD2* | Radical S-adenosyl methionine domain containing 2 | *RSAD2* plays a role in cellular antiviral response and innate immune signaling by interference in the secretory pathway, inhibition of viral RNA replication, binding to viral proteins and dysregulation of cellular lipid metabolism. |
| *RGCC* | Regulator of cell cycle | *RGCC* can regulate cell cycle progression induced by p53 in response to DNA damage, or by sublytic levels of complement system proteins. |
| *SLBP* | Stem-loop binding protein | SLBP protein can regulate cell cycle. It binds to the stem-loop structure in replication-dependent histone mRNAs. The stem-loop structure controls the transport, translation and stability of histone mRNAs. |
| *DRAM1* | DNA-damage regulated autophagy modulator 1 | *DRAM1* is regulated as part of the p53 tumor suppressor pathway. DRAM1 protein is required for the induction of autophagy. |
| *IFIT5* | Interferon-induced protein with tetratricopeptide repeats 5 | IFIT5 protein is associate with the innate immune response, and it has a broad and adaptable RNA structure recognition important for RNA recognition specificity in antiviral defense. |
| *SAMD9L* | Sterile alpha motif domain-containing protein 9-like | SAMD9L protein, a tumor suppressor, plays a key role in cell proliferation and the innate immune response to viral infection. |
| *KAT2A* | Lysine acetyltransferase 2a | KAT2A functions primarily as a transcriptional activator. It also functions as a repressor of NFκB by promoting ubiquitination of the NFκB subunit RELA in a HAT-independent manner. |
| *SERPINB2* | Serpin family b member 2 | *SERPINB2* can elevate the cytosolic Ca^2+^ and transcription receptor-mediated HIF regulation. It relates to serine-type endopeptidase inhibitor activity. |
| *OASL* | 2'-5'-oligoadenylate synthetase like | *OASL* relates to interferon gamma signaling, innate immune system, RNA binding and double-stranded RNA binding. |
| *TLR3* | Toll-like receptor 3 | TLR3 protein plays a fundamental role in pathogen recognition and activation of innate immunity. It recognizes pathogen-associated molecular patterns (PAMPs) that are expressed on infectious agents, and mediate the production of cytokines necessary for the development of effective immunity. |
| *NT5C3B* | 5'-nucleotidase, cytosolic iiib | *NT5C3B* is link to deadenylation-dependent mRNA decay, nucleotide binding and 5'-nucleotidase activity. |
| *IFIH1* | Interferon induced with helicase c domain 1 | *IFIH1* encodes MDA5 protein which is an intracellular sensor of viral RNA that triggers the innate immune response. MDA5 has also been shown to play an important role in enhancing natural killer cell function in malaria infection. |
| *ZC3HAV1* | Zinc finger CCCH-type antiviral protein 1 | *ZC3HAV1* is associated with nervous system development and differentiation through its involvement in the up-regulation of a number of genes which are required for neurogenesis. |
| *EPSTI1* | Epithelial-stromal interaction protein 1 | *EPSTI1* protein interacts with the amino terminal region of the valosin containing protein gene product, resulting in the nuclear translocation of the NFκB subunit 1 gene product, and activation of target genes. |
| *CMPK2* | Cytidine/uridine monophosphate kinase 2 | *CMPK2* encodes one of the enzymes in the nucleotide synthesis salvage pathway that may participate in terminal differentiation of monocytic cells. |
| *TOR4A* | Torsin family 4 member A | *TOR4A* is response to elevated platelet cytosolic Ca^2+^. |
| *ZNFX1* | Zinc finger, NFX1-type containing 1 | ZNFX1 functions as a dsRNA sensor to initiate antiviral responses. |
| *STAT1* | Signal transducer and activator of transcription 1 | *STAT1* may be implicated in interstrand DNA cross-link repair and in the maintenance of normal chromosome stability. |
| *CPO* | Carboxypeptidase O | *CPO* related to metallocarboxypeptidase activity. A paralog of *CPO* is *CPA6.* |
| *FAM234A* | Family with sequence similarity 234 member A | *FAM234A* is associated with glucosephosphate dehydrogenase deficiency. |
| *MYD88* | Myeloid differentiation primary response protein myd88 | *MYD88* is involved in the TLR and IL1R signaling pathway in the innate immune response. It participates in the innate immune response to microbial agents. |
| *SLC2A5* | Solute carrier family 2 member 5 | *SLC2A5* is a fructose transporter responsible for fructose uptake by the small intestine. |
| *USP18* | Ubiquitin carboxyl-terminal hydrolase | Mice lacking *USP18* gene are hypersensitive to interferon, suggesting a function of this protein in downregulating interferon responses, independent of its isopeptidase activity towards ISG15. |
| *TMEM86A* | Transmembrane protein 86A | TMEM86A is associated with autosomal recessive congenital ichthyosis. An important paralog of this gene is *TMEM86B*. |
| *MX1* | Interferon-induced GTP-binding protein mx | *MX1* participates in the cellular antiviral response. MX1 protein is induced by type I and type II interferons and antagonizes the replication process of several different RNA and DNA viruses. |
| *LIPA* | Lysosomal acid lipase/cholesteryl ester hydrolase | *LIPA* encodes lipase A which functions in the lysosome to catalyze the hydrolysis of cholesteryl esters and triglycerides. |
| *MAN1A1* | Mannosidase, alpha, class 1A, member 1 | *MAN1A1* relates to the transport to the golgi and subsequent modification. It also links to calcium ion binding and mannosidase activity. |
| *CDHR2* | Cadherin-related family member 2 | *CDHR2* is a member of the protocadherin family, which represents a subset of the larger cadherin superfamily. It functions as calcium-dependent cell-cell adhesion molecules. |
| *TMEM123* | Transmembrane protein 123 | *TMEM123* encodes a highly glycosylated transmembrane protein with a high content of threonine and serine residues in its extracellular domain. This gene product is proposed to function as a cell surface receptor that mediates cell death. |
| *SLC25A22* | Solute carrier family 25, member 22 | *SLC25A22* encodes a mitochondrial glutamate carrier. *SLC25A22* Mutations are associated with early infantile epileptic encephalopathy. |
| *CLIC5* | Chloride intracellular channel 5 | *CLIC5* encodes a member of the chloride intracellular channel (CLIC) family of chloride ion channels. CLIC5 may play a role in multiple processes including myoblast proliferation, glomerular podocyte and endothelial cell maintenance. |
| *RAB27A* | Member RAS oncogene family | RAB27A belongs to the small GTPase superfamily, Rab family. RAB27A may be involved in protein transport and small GTPase mediated signal transduction. |
| *GAB2* | Grb2 associated binding protein 2 | *GAB2* acts as adapters for transmitting various signals in response to stimuli. GAB2 is the principal activator of phosphatidylinositol-3 kinase in response to activation of the high affinity IgE receptor. |
| *NFKBIZ* | NFKB inhibitor zeta | NFKBIZ protein is a member of the ankyrin-repeat family and is induced by lipopolysaccharide (LPS). It may play a role in inflammatory responses to LPS by their interaction with NFκB proteins through ankyrin-repeat domains. |
| *KMT2E* | Lysine (K)-specific methyltransferase 2E | KMT2E protein is a member of the myeloid/lymphoid family and encodes a protein with an N-terminal PHD zinc finger and a central SET domain. Overexpression of KMT2E protein inhibits cell cycle progression. |
| *LRCH2* | Leucine-rich repeats and calponin homology (CH) domain containing 2 | *LRCH2* encodes the leucine-rich repeat and calponin homology domain-containing protein family which are conserved across animal species, and functions as a cytoskeletal scaffolding protein. |
| *PNISR* | PNN-interacting serine/arginine-rich protein | PNISR is related to RNA binding. |
| *RASD1* | Dexamethasone-induced 1 | *RASD1* encodes a member of the Ras superfamily of small GTPases and is induced by dexamethasone. *RASD1* may play a role in dexamethasone-induced alterations in cell morphology, growth and cell-extracellular matrix interactions. |
| *IL8L2* | Interleukin-8 precursor; Interleukin-8 | *IL8L2* may be an autocrine factor that promotes the growth of fibroblasts and is involved in the neoplastic transformation of fibroblasts by v-Src. *IL8L2* is chemotactic for peripheral blood mononuclear cells as well as for heterophils |
| *STK38L* | Serine/threonine kinase 38 like | STK38L is relate to transferase activity, transferring phosphorus-containing groups and protein tyrosine kinase activity. STK38L is involved in the regulation of structural processes in differentiating and mature neuronal cells. |

Supplemental Table 5. The top 20 up-regulated genes in the jejuna of LPC500 group compared to the control

| Gene symbol | Log_2_FC | *P* Value | Description |
| --- | --- | --- | --- |
| *RSAD2* | 2.82 | 8.30E-03 | Radical S-adenosyl methionine domain containing 2 |
| *RGCC* | 2.79 | 5.89E-03 | Regulator of cell cycle |
| *SLC25A48* | 2.23 | 2.42E-02 | Solute carrier family 25 member 48 |
| *SLBP* | 2.07 | 1.35E-02 | Stem-loop binding protein |
| *DRAM1* | 2.02 | 3.31E-03 | DNA damage regulated autophagy modulator 1 |
| *IFIT5* | 2.01 | 7.45E-03 | Interferon-induced protein with tetratricopeptide repeats 5 |
| *AREG* | 1.92 | 4.85E-03 | Amphiregulin |
| *PON2* | 1.89 | 5.91E-04 | Paraoxonase 2 |
| *SAMD9L* | 1.84 | 6.91E-03 | Sterile alpha motif domain containing 9-like |
| *KAT2A* | 1.8 | 8.80E-04 | K(lysine) acetyltransferase 2A |
| *SERPINB2* | 1.8 | 7.26E-03 | Serpin peptidase inhibitor, clade B (ovalbumin), member 2 |
| *TRANK1* | 1.8 | 1.39E-02 | Tetratricopeptide repeat and ankyrin repeat containing 1 |
| *OASL* | 1.79 | 4.44E-04 | 2'-5'-oligoadenylate synthetase-like |
| *TLR3* | 1.76 | 2.99E-05 | Toll-like receptor 3 |
| *NT5C3B* | 1.68 | 3.23E-04 | 5'-nucleotidase, cytosolic IIIB |
| *IFIH1* | 1.64 | 4.20E-03 | Interferon induced, with helicase C domain 1 |
| *GCG* | 1.63 | 4.88E-02 | Glucagon |
| *PLK3* | 1.62 | 1.47E-02 | Polo-like kinase 3 |
| *FAM188A* | 1.6 | 1.50E-02 | Family with sequence similarity 188 member A |
| *PTH1R* | 1.59 | 3.01E-02 | Parathyroid hormone 1 receptor |

Supplemental Table 6. The top 20 down-regulated genes in the jejuna of LPC500 group compared to the control

| Gene symbol | Log_2_FC | *P* Value | Description |
| --- | --- | --- | --- |
| *CYP2C8* | -2.81 | 1.97E-02 | Cytochrome P450, family 2, subfamily C, polypeptide 8 |
| *LOC107052494* | -2.06 | 1.15E-02 | Gtpase IMAP family member 7-like |
| *LOC427933* | -2.05 | 1.86E-03 | Sulfotransferase 6B1-like |
| *STK38L* | -2.01 | 2.94E-05 | Serine/threonine kinase 38 like |
| *FGG* | -1.93 | 1.44E-02 | Fibrinogen gamma chain |
| *ABCB11* | -1.88 | 4.40E-02 | ATP-binding cassette, sub-family B (MDR/TAP), member 11 |
| *IL8L2* | -1.79 | 2.40E-02 | Interleukin 8-like 2 |
| *LOC419204* | -1.79 | 3.39E-02 | Similar to junctophilin type 2 |
| *SOGA2* | -1.69 | 1.20E-03 | SOGA family member 2 |
| *LOC769185* | -1.63 | 1.79E-02 | Breast carcinoma-amplified sequence 1-like |
| *PIGR* | -1.6 | 6.34E-04 | Polymeric immunoglobulin receptor |
| *FBXO22* | -1.6 | 2.24E-02 | F-box protein 22 |
| *KCNE3* | -1.5 | 3.79E-02 | Potassium voltage-gated channel subfamily E regulatory subunit 3 |
| *LOC420486* | -1.44 | 1.02E-02 | Mycocerosic acid synthase-like |
| *MLN* | -1.36 | 5.73E-03 | Motilin |
| *TMSB15B* | -1.34 | 3.45E-03 | Thymosin beta 15B |
| *FAM169B* | -1.29 | 1.73E-03 | Family with sequence similarity 169 member B |
| *LOC776526* | -1.29 | 1.99E-02 | Similar to CRP-ductin-alpha |
| *RASD1* | -1.28 | 2.86E-03 | RAS, dexamethasone-induced 1 |
| *PNISR* | -1.25 | 1.41E-02 | PNN-interacting serine/arginine-rich protein |

Supplemental Table 7. The top 20 up-regulated genes in the jejuna of LPC1000 group compared to the control

| Gene symbol | Log_2_FC | *P* Value | Description |
| --- | --- | --- | --- |
| *RSAD2* | 2.48 | 6.51E-03 | Radical S-adenosyl methionine domain containing 2 |
| *SERPINB2* | 2.42 | 8.40E-03 | Serpin peptidase inhibitor, clade B (ovalbumin), member 2 |
| *CA7* | 2.32 | 3.16E-03 | Carbonic anhydrase VII |
| *ASAH2* | 2.32 | 1.55E-02 | N-acylsphingosine amidohydrolase (non-lysosomal ceramidase) 2 |
| *LCT* | 1.92 | 4.57E-02 | Lactase |
| *RGCC* | 1.89 | 3.58E-02 | Regulator of cell cycle |
| *ASB5* | 1.84 | 1.24E-02 | Ankyrin repeat and SOCS box containing 5 |
| *CD36* | 1.84 | 3.35E-02 | CD36 molecule |
| *CYP1A1* | 1.83 | 2.38E-02 | Cytochrome P450, family 1, subfamily A, polypeptide 1 |
| *TFPI2* | 1.82 | 1.49E-03 | Tissue factor pathway inhibitor 2 |
| *MYH11* | 1.81 | 5.92E-03 | Myosin, heavy chain 11, smooth muscle |
| *COL3A1* | 1.8 | 6.34E-04 | Collagen, type III, alpha 1 |
| *CYP4V2* | 1.8 | 1.91E-03 | Cytochrome P450 family 4 subfamily V member 2 |
| *SERPINE2* | 1.79 | 9.91E-03 | Serpin peptidase inhibitor, clade E (nexin, plasminogen activator inhibitor type 1), member 2 |
| *REG4* | 1.73 | 3.18E-02 | Regenerating islet-derived family, member 4 |
| *CDH11* | 1.71 | 2.18E-03 | Cadherin 11, type 2, OB-cadherin (osteoblast) |
| *ALDH1A3* | 1.71 | 1.31E-02 | Aldehyde dehydrogenase 1 family, member A3 |
| *AQP5* | 1.7 | 1.96E-03 | Aquaporin 5 |
| *TGFBI* | 1.7 | 3.45E-03 | Transforming growth factor, beta-induced, 68kda |
| *HSD3B7* | 1.63 | 4.53E-03 | Hydroxy-delta-5-steroid dehydrogenase, 3 beta- and steroid delta-isomerase 7 |

Supplemental Table 8. The top 20 down-regulated genes in the jejuna of LPC1000 group compared to the control

| Gene symbol | Log_2_FC | *P* Value | Description |
| --- | --- | --- | --- |
| *AVD* | -2.60 | 3.03E-02 | Avidin |
| *IL8L2* | -2.44 | 1.92E-03 | Interleukin 8-like 2 |
| *LOC107052494* | -2.06 | 3.99E-02 | Gtpase IMAP family member 7-like |
| *LOC420486* | -1.8 | 1.29E-03 | Mycocerosic acid synthase-like |
| *STK38L* | -1.78 | 2.17E-03 | Serine/threonine kinase 38 like |
| *IL8L1* | -1.63 | 6.26E-03 | Interleukin 8-like 1 |
| *SIPA1L2* | -1.63 | 6.94E-03 | Signal-induced proliferation-associated 1 like 2 |
| *SOCS3* | -1.63 | 1.37E-02 | Suppressor of cytokine signaling 3 |
| *CHKA* | -1.54 | 2.54E-02 | Choline kinase alpha |
| *STEAP1* | -1.34 | 4.73E-03 | Six transmembrane epithelial antigen of the prostate 1 |
| *CPT1A* | -1.31 | 6.27E-03 | Carnitine palmitoyltransferase 1A (liver) |
| *SLC35B3* | -1.29 | 1.37E-02 | Solute carrier family 35 (adenosine 3'-phospho 5'-phosphosulfate transporter), member B3 |
| *ZNF711* | -1.28 | 6.96E-04 | Zinc finger protein 711 |
| *KMT2E* | -1.25 | 3.36E-03 | Lysine (K)-specific methyltransferase 2E |
| *GAB2* | -1.24 | 3.34E-03 | GRB2-associated binding protein 2 |
| *PNISR* | -1.23 | 1.34E-02 | PNN-interacting serine/arginine-rich protein |
| *ZPLD1* | -1.19 | 6.57E-03 | Zona pellucida-like domain containing 1 |
| *LOC769704* | -1.15 | 2.08E-02 | Fatty acyl-coa hydrolase precursor, medium chain-like |
| *FAS* | -1.14 | 1.11E-03 | Fas cell surface death receptor |
| *LOC420485* | -1.13 | 1.97E-02 | Uncharacterized LOC420485 |

Supplemental Table 9. The top 20 up-regulated genes in the jejuna of LPC1000 group compared to LPC500 group

| Gene symbol | Log_2_FC | *P* Value | Description |
| --- | --- | --- | --- |
| *CYP2C8* | 3.44 | 4.85E-03 | Cytochrome P450, family 2, subfamily C, polypeptide 8 |
| *CYP1A4* | 2.78 | 7.97E-04 | Cytochrome P450 1A4 |
| *CYP1A1* | 2.2 | 5.35E-03 | Cytochrome P450, family 1, subfamily A, polypeptide 1 |
| *COL3A1* | 2.16 | 2.52E-02 | Collagen, type III, alpha 1 |
| *MGP* | 2.14 | 1.32E-02 | Matrix Gla protein |
| *MYLK* | 2.11 | 3.04E-02 | Myosin light chain kinase |
| *MMP2* | 2.06 | 2.52E-02 | Matrix metallopeptidase 2 (gelatinase A, 72kda gelatinase, 72kda type IV collagenase) |
| *MPZL2* | 1.96 | 6.71E-04 | Myelin protein zero like 2 |
| *GIP* | 1.95 | 2.46E-02 | Gastric inhibitory polypeptide |
| *SERPINE2* | 1.94 | 1.09E-02 | Serpin peptidase inhibitor, clade E (nexin, plasminogen activator inhibitor type 1), member 2 |
| *COL6A1* | 1.87 | 3.25E-02 | Collagen, type VI, alpha 1 |
| *AQP5* | 1.86 | 1.44E-03 | Aquaporin 5 |
| *IGFBP7* | 1.86 | 3.14E-02 | Insulin like growth factor binding protein 7 |
| *IYD* | 1.83 | 7.45E-03 | Iodotyrosine deiodinase |
| *MYH11* | 1.83 | 2.06E-02 | Myosin, heavy chain 11, smooth muscle |
| *CYP2C45* | 1.82 | 1.02E-02 | Cytochrome P-450 2C45 |
| *ALDH1A3* | 1.82 | 3.72E-02 | Aldehyde dehydrogenase 1 family, member A3 |
| *LOC427292* | 1.81 | 4.61E-03 | S-adenosylmethionine synthase isoform type-2-like |
| *DCN* | 1.77 | 3.96E-02 | Decorin |
| *CA7* | 1.70 | 5.79E-03 | Carbonic anhydrase VII |

Supplemental Table 10. The top 20 down-regulated genes in the jejuna of LPC1000 group compared to LPC500 group

| Gene symbol | Log_2_FC | *P* Value | Description |
| --- | --- | --- | --- |
| *SLC10A2* | -4.07 | 3.87E-03 | Solute carrier family 10 (sodium/bile acid cotransporter), member 2 |
| *FABP6* | -3.76 | 4.96E-03 | Fatty acid binding protein 6 |
| *UPP1* | -3.14 | 1.06E-02 | Uridine phosphorylase 1 |
| *SLC25A48* | -2.33 | 2.26E-02 | Solute carrier family 25 member 48 |
| *FOXD1* | -2.28 | 3.26E-02 | Forkhead box D1 |
| *KCNJ15* | -2.22 | 5.62E-03 | Potassium inwardly-rectifying channel, subfamily J, member 15 |
| *KCNJ16* | -2.11 | 2.10E-02 | Potassium voltage-gated channel subfamily J member 16 |
| *TMEM252* | -1.97 | 2.49E-02 | Transmembrane protein 252 |
| *PTH1R* | -1.76 | 1.91E-02 | Parathyroid hormone 1 receptor |
| *SUSD2* | -1.69 | 4.93E-02 | Sushi domain containing 2 |
| *WFDC2* | -1.66 | 1.51E-03 | WAP four-disulfide core domain 2 |
| *PON2* | -1.62 | 7.91E-03 | Paraoxonase 2 |
| *TC2N* | -1.53 | 4.53E-02 | Tandem C2 domains, nuclear |
| *MCOLN2* | -1.41 | 2.86E-02 | Mucolipin 2 |
| *SATB2* | -1.36 | 2.00E-02 | SATB homeobox 2 |
| *FRMD3* | -1.32 | 4.57E-02 | FERM domain containing 3 |
| *GCG* | -1.31 | 2.84E-02 | Glucagon |
| *FAM3D* | -1.29 | 1.94E-02 | Family with sequence similarity 3, member D |
| *SEMA6D* | -1.28 | 2.27E-02 | Sema domain, transmembrane domain (TM), and cytoplasmic domain, (semaphorin) 6D |
| *LOC426155* | -1.22 | 4.56E-02 | Uncharacterized gene |

Supplemental Table 11. Hub genes involved in the effects of LPC on chicken jejuna

| Genes symbol | Full name | Functions |
| --- | --- | --- |
| *RSAD2* | Radical S-adenosyl methionine domain containing 2 | RSAD2 protein plays a role in cellular antiviral response and innate immune signaling by interference in the secretory pathway, inhibition of viral RNA replication, binding to viral proteins and dysregulation of cellular lipid metabolism. |
| *OASL* | 2'-5'-oligoadenylate synthetase-like | OASL protein is related to interferon gamma signaling, innate immune system, RNA binding and double-stranded RNA binding. |
| *EPSTI1* | Epithelial-stromal interaction protein 1 | EPSTI1 protein interacts with the amino terminal region of the valosin containing protein gene product, resulting in the nuclear translocation of the nuclear factor kappa B subunit 1 gene product, and activation of target genes. |
| *CMPK2* | Cytidine monophosphate (UMP-CMP) kinase 2 | *CMPK2* encodes one of the enzymes in the nucleotide synthesis salvage pathway that may participate in terminal differentiation of monocytic cells. |
| *IFIH1* | interferon induced with helicase C domain 1 | *IFIH1* encodes MDA5 protein which is an intracellular sensor of viral RNA that triggers the innate immune response. MDA5 has also been shown to play an important role in enhancing natural killer cell function in malaria infection. |
| *IFIT5* | Interferon-induced protein with tetratricopeptide repeats 5 | IFIT5 protein is associated with the innate immune response, and it has a broad and adaptable RNA structure recognition important for RNA recognition specificity in antiviral defense. |
| *USP18* | Ubiquitin carboxyl-terminal hydrolase | Mice lacking *USP18* gene are hypersensitive to interferon, suggesting a function of this protein in downregulating interferon responses, independent of its isopeptidase activity towards ISG15. |
| *MX1* | Interferon-induced gtp-binding protein mx | *MX1* participates in the cellular antiviral response. MX1 protein is induced by type I and type II interferons and antagonizes the replication process of several different RNA and DNA viruses. |
| *STAT1* | signal transducer and activator of transcription 1 | *STAT1* may be implicated in interstrand DNA cross-link repair and in the maintenance of normal chromosome stability. |
| *SAMD9L* | Sterile alpha motif domain-containing protein 9-like | SAMD9L protein, a tumor suppressor, plays a key role in cell proliferation and the innate immune response to viral infection. |
| *ZNFX1* | Zinc finger, NFX1-type containing 1 | ZNFX1 protein functions as a dsRNA sensor to initiate antiviral responses through MAVS. |
| *TLR3* | Toll-like receptor 3 | TLR3 protein plays a fundamental role in pathogen recognition and activation of innate immunity. It recognizes pathogen-associated molecular patterns (PAMPs) that are expressed on infectious agents, and mediate the production of cytokines necessary for the development of effective immunity. |
